# Developing sustainable patient and public involvement in mesothelioma research: multi-method exploration with researchers, patients, carers, and patient organisations

**DOI:** 10.1186/s40900-023-00426-5

**Published:** 2023-03-25

**Authors:** Afrodita Marcu, Fiona McGregor, Bernadette Egan, Kate Hill, Tim Cook, Anne Arber

**Affiliations:** 1 School of Health Sciences, Faculty of Health and Medical Sciences, University of Surrey, Guildford, Surrey, GU2 7YH UK; 2 School of Biosciences, Faculty of Health and Medical Sciences, University of Surrey, Guildford, UK; 3 School of Medicine, Faculty of Medicine and Health, University of Leeds, Leeds, UK; 4 Runnymede, UK

**Keywords:** Cancer, Mesothelioma, Patient and public involvement, Qualitative

## Abstract

**Background:**

Rare diseases where prognosis is poor provide limited scope for patient and public involvement (PPI). One such disease is mesothelioma, a cancer of the lung pleura or of the peritoneum caused by exposure to asbestos, where PPI is poorly documented. We undertook to explore how PPI could be facilitated in mesothelioma research.

**Methods:**

An online survey with mesothelioma researchers (n = 23) assessed the perceived benefits and challenges of PPI in mesothelioma. Six online workshops and thirteen in-depth interviews with patients and the public explored their views on how PPI could be increased in mesothelioma and their motivations to become PPI representatives in the future. The survey data were analysed using descriptive statistics and the interviews, using Thematic Analysis.

**Results:**

In the survey, 26% (n = 6) of the researchers did not include PPI in their research, while 74% (n = 17) did, finding it most beneficial at the stages of applying for funding and dissemination. The main perceived benefits of PPI were clarifying the research question and outcome measures, making research more credible and relevant to patients’ needs, and increasing its impact. The main perceived challenges to PPI were the general poor prognosis in mesothelioma, and funding timescales which hindered timely recruitment of PPI representatives. The analysis of the interviews with the patients and public revealed three main themes: “Motivations to become a PPI representative in the future”, “Understanding the nature of PPI during the project”, and “Perceived challenges to PPI in mesothelioma”. Altruism and the need for hope were the main reasons to wish to become involved in PPI in the future. For many participants, the project proved to be a journey of understanding the nature of PPI, a concept that was not easy to grasp from the start. The participants perceived certain barriers to PPI such as high symptom burden in mesothelioma, the abstract concept of PPI, and the use of scientific language.

**Conclusions:**

The present research provides a detailed picture of the benefits and challenges of PPI in mesothelioma. We recommend long-term engagement with mesothelioma support groups so that researchers achieve meaningful and sustainable PPI in mesothelioma research.

**Supplementary Information:**

The online version contains supplementary material available at 10.1186/s40900-023-00426-5.

## Abstract (summary)

Patient and public involvement (PPI) in research means research that is done ‘with’ or ‘by’ the public, not ‘to’, ‘about’, or ‘for’ them. Involving patients, family caregivers or the public (e.g. coordinators of patient organisations) in research activities means that they contribute to how research is designed, conducted, or disseminated. However, some diseases where patients have a short prognosis after diagnosis make it harder for patients or their family members to get involved. In this project we explored the perspectives of patients, public, and researchers on how PPI could be increased and maintained in research on mesothelioma—a rare cancer of the lining of the lung or of the peritoneum caused by exposure to asbestos. We conducted an online survey with mesothelioma researchers, and they indicated a number of PPI benefits but also challenges such as finding people living with mesothelioma well enough to participate. We also conducted six online workshops and thirteen interviews with patients, family members and coordinators of mesothelioma patient organisations—these were not PPI representatives but participants in research. All participants in the project were motivated by the wish to enhance outcomes for other patients. Their understanding of PPI improved during the project and they saw a number of challenges to others becoming involved in PPI, such as its abstract nature and the use of scientific language. We recommend that researchers engage long-term with mesothelioma support groups so that they have opportunities to explain what PPI means and involve people affected by mesothelioma in research.

**Supplementary Information:**

The online version contains supplementary material available at 10.1186/s40900-023-00426-5.

## Introduction

Rare diseases with a high symptom burden and a short prognosis can make it difficult for patients and/or their family members to be involved as patient and public involvement representatives in health research. As defined by the National Institute for Health & Care Research, patient and public involvement (PPI) in research means research that is done ‘with’ or ‘by’ the public, not ‘to’, ‘about’ or ‘for’ them [1]. It means that patients or other people with relevant experience contribute to how research is designed, conducted and disseminated. Research which involves PPI is carried out within an active partnership with patients, carers and lay members of the public (i.e., service users and ordinary citizens) that influences and shapes research. However, the nature of some diseases can limit the potential to involve patients and family members. In this paper we take the example of a rare form of cancer, mesothelioma, where patients have a short prognosis, to detail various stakeholders’ perspectives on PPI and suggest ways for how researchers can overcome barriers to PPI in mesothelioma research.

Malignant pleural mesothelioma is a cancer of the lung pleura while peritoneal mesothelioma is a cancer of the peritoneum (the tissue lining the inside of the abdomen). Rarer forms of mesothelioma can also involve pericardial or testicular tissue. Asbestos is the major risk factor for mesothelioma and the disease can develop up to 50 years after exposure [2]. About 2700 people are diagnosed with mesothelioma annually in the UK [3]. Symptoms of pleural mesothelioma, the most common type, usually include shortness of breath (dyspnea), chest pain, fatigue, weight loss, a persistent cough, heavy sweating at night, difficulty swallowing, and a hoarse voice [4]. Symptoms of peritoneal mesothelioma include abdominal pain, swelling in the abdomen, loss of appetite, weight loss, diarrhoea or constipation [5]. Symptomatic presentation for mesothelioma is typically with advanced disease, and median survival is 8–14 months after diagnosis [6], although some patients do survive for longer.

The experience of being diagnosed with mesothelioma is known to be psychologically and emotionally distressing, with a high symptom burden experienced by patients alongside a severely reduced lifespan and poor prognosis [7–10]. Given the burden of the disease experienced by patients and their families, and the relatively short prognosis after diagnosis, it can be difficult for researchers to involve people with mesothelioma and/or their family caregivers in PPI activities. However, there is evidence that increasing patient involvement in mesothelioma research engenders hope for the future in those involved, through knowledge of research agendas [11–14]. Nonetheless, successful PPI strategies are still lacking despite such involvement being regarded as good practice in advancing good quality health care, including cancer care [15–18].

The inclusion of PPI in mesothelioma research is relatively low and insufficiently documented. While some studies mention involving patient representatives [6, 19, 20], few detail how they have involved patients throughout the research process. To address this gap, we undertook to explore how PPI could be developed in mesothelioma research. The present project built on previous research that explored how patient and public engagement can be widened in mesothelioma research [21]. The aims of the present research were two-fold: to explore the barriers and facilitators to PPI in mesothelioma, and to develop best practice for PPI in mesothelioma in collaboration with patients, carers, and patient organisations. In this article we detail the perceived barriers and facilitators to PPI in mesothelioma from different stakeholder perspectives and propose recommendations for how to create effective PPI in mesothelioma research.

## Methods

This project comprised two studies which were conducted in parallel: a survey with mesothelioma researchers to explore the breadth and depth of PPI in mesothelioma research and the perceived benefits and challenges of including PPI; and six workshops and 13 individual interviews with people living with mesothelioma, family carers, bereaved family members and coordinators of mesothelioma/asbestos patient organisations to explore perceived barriers and facilitators to participating in PPI in mesothelioma research. All the data were collected online due to the COVID-19 pandemic.

## Design

### Study 1: the researchers’ perspective on PPI in mesothelioma

An online survey was conducted with researchers from the Mesothelioma Research Network (MRN) to scope the extent of PPI in mesothelioma research. The survey was conducted using the online platform Qualtrics [22] and the survey items were informed by studies on the barriers and facilitators to PPI in health research [23, 24]. The survey explored reasons for including or not including PPI, perceived challenges and perceived benefits of including PPI in mesothelioma research. If the respondents indicated that they currently included PPI, they were asked to indicate the stages of the research process where they usually included PPI, e.g. study design [23], the benefits they associated with PPI, e.g. improved credibility of research proposals [24], and, from their experience, the main drawbacks and challenges when including PPI in research (open-ended answers). If the survey participants did not currently include PPI, they were redirected to questions eliciting reasons for not including PPI, perceived costs of PPI, and perceptions of research stages where PPI might be beneficial. Answers were measured on 5-point Likert scales ranging from ‘strongly agree’ to ‘strongly disagree’, such as for questions pertaining to perceived costs of PPI, perceived benefits of PPI at certain stages of the research process, e.g. data collection. Answers to questions pertaining to how often the researchers included PPI at various stages of the research process were measured on 5-point Likert scales from ‘always’ to ‘never’, with the additional option of ‘don’t know/ N/A’. Answers to the question “which stage of the research do you find PPI most beneficial for your own research?” were measured on a 5-point Likert scale from ‘extremely beneficial’ to ‘not at all beneficial’, with the additional option of ‘don’t know/ N/A’. Please see Table 1 for an illustration of the items and the scales used in the survey.

**Table 1:** Sample items and respective scales from the survey with mesothelioma researchers

Patient and Public Involvement & Engagement (PPI/E) activities at the agenda setting stage	Always	Usually	Sometimes	Rarely	Never	N/A
*How often do you include the following PPI/E tasks and activities when setting your research agenda?*						
1. Identifying or generating research topics or questions	□	□	□	□	□	□
2. Prioritising topics for research	□	□	□	□	□	□
3. Providing a patient perspective on outcomes that are important to patients and their families	□	□	□	□	□	□

	Extremely beneficial	Quite beneficial	Neither beneficial nor unhelpful	Somewhat Beneficial	Not at all beneficial	Don’t know, N/A
*At which stage of the research do you find PPI/E most beneficial for your own research?*						
1. Agenda setting	□	□	□	□	□	□
2. Applying for funding	□	□	□	□	□	□
3. Dissemination	□	□	□	□	□	□

Due to the small number of respondents, ‘agree’ and ‘strongly agree’ answers were recategorized as ‘agree’, while ‘disagree’ and ‘strongly disagree’ were recategorized as ‘disagree’ similarly to previous research [25]. Similarly, ‘always’ and ‘usually’ answers were recategorized as ‘usually’, while ‘rarely’ and ‘never’ as ‘never’. For other questions, ‘extremely beneficial’ and ‘quite beneficial’ answers were recategorized as ‘beneficial’, and ‘somewhat beneficial’ and ‘not at all beneficial’, as ‘not beneficial’, respectively.

### Study 2: The patient and public perspective on PPI in mesothelioma

The second study involved a series of workshops where we consulted with patients, carers, and the public. These three broad categories were represented in our study as such: the ‘patients’ were people living with mesothelioma (although they were recruited via support groups and not from clinical settings); the ‘carers’ were their family members and bereaved family members; and the ‘public’ were coordinators of mesothelioma patient organisations. We chose the latter as they are familiar with the support needs of people living with or affected by mesothelioma. In these workshops we explored the perceived barriers and facilitators to participating in PPI and the possibility to create a sustainable group of PPI representatives to be ready to participate in PPI activities in mesothelioma research. We drew on the principles of co-production [26], as co-production is part of the spectrum of patient and public involvement [27].Thus, we aimed to: create a forum (albeit an online one) where all participants worked together to achieve a joint purpose and shared understanding; include a wide range of perspectives and skills; and make everyone feel valued for their knowledge and experience. The workshops were facilitated by one member of the research team who was experienced in group facilitation, with additional facilitation from a researcher with expertise in PPI and mesothelioma research who was a trustee of the mesothelioma charity funding the current research. A series of individual interviews were subsequently conducted with the workshop participants to elicit their experience during the workshops and to understand their personal journey of learning about PPI, including their thoughts on the best ways to sustain PPI in mesothelioma research.

## Participants and procedure

### Study 1: the researchers’ perspective on PPI in mesothelioma

The participants in the survey were recruited from among members of the Mesothelioma Research Network. The members come from various professional backgrounds (clinicians, academics, researchers) with various specialities (e.g. respiratory medicine, epidemiology, histopathology, clinical oncology). An invitation to take part in the study with a link to the survey was sent via key contacts at the British Lung Foundation (now Asthma + Lung UK) to the mesothelioma researchers on their database. The participants were informed that the survey explored whether mesothelioma researchers involve and/or engage patients, carers, and the public in their research and to what extent they do so. The participants were informed at the beginning of the survey that they can withdraw from the survey at any point without prejudice. Upon completion of the survey the participants had the option to enter a prize draw for Love2Shop vouchers worth £100 as compensation for their time. The survey was piloted with a small group of MRN members (n = 4), while twenty participants completed the main survey. A few minor changes were made following the pilot study, e.g. enabling multiple answers to the questions pertaining to the respondents’ main types of research and to the respondents’ current type of research on mesothelioma. The responses from the MRN members who piloted the survey were included in the data analysis. One participant who took part in the pilot survey also completed the main survey, therefore only their responses to the main survey were considered for the analysis.

### Study 2: the patient and public perspective on PPI in mesothelioma

Participants were recruited through asbestos and mesothelioma support charities, with flyers for the study being distributed to members with approval from the respective charities. Previous involvement in PPI activities was not an exclusion criterion but we aimed to include as many people as possible who were new to PPI. Recruitment took place during the COVID-19 pandemic in 2020, and thus it was carried out remotely at patient organisations coffee mornings held over Zoom. There were 23 expressions of interest from the members of various asbestos and mesothelioma patient organisations. Participant information sheets and consent forms were sent by email to members who expressed an interest to take part. The participants were informed that they could withdraw from the study at any point without prejudice. Overall, 14 participants were recruited in the period October 2020–January 2021. Email contact was sustained throughout the project both on a group and individual basis by one member of the research team. Additional patients, co-ordinators of patient organisations and researchers were invited to take part in the workshops by one researcher who was a trustee of the mesothelioma charity funding the current research.

Seven people living with mesothelioma, age range 59–79, agreed to take part in the study. Of these, five had been living with pleural mesothelioma for a number of years, ranging from 2 to 12 years post diagnosis; one had been diagnosed with peritoneal mesothelioma a few months earlier; and another one sadly passed away during the project, eight months after being diagnosed with pleural mesothelioma. Five family members, of which four were bereaved, also participated in the workshops /interviews. We viewed the bereaved family members who participated in our research as fulfilling the role of sharing their experiential knowledge of caring for someone diagnosed with and treated for mesothelioma [28]. Three workers at patient organisations supporting people with a range of asbestos-related diseases, including mesothelioma, pleural thickening asbestosis, and lung cancer also attended some of the workshops and some of these took part in the interviews. Six workshops, lasting approximately one hour, were held monthly over Zoom from October 2020 to March 2021, covering topics such as: introduction and scoping; explaining why the lived experience as people—or family members of people—diagnosed with and treated for mesothelioma is of value to mesothelioma researchers; explaining the concept of PPI and introduction to online PPI training by inviting attendees to complete the European Patient Ambassador Programme module online [29]; presentations from two mesothelioma researchers; role of PPI in dissemination; discussion of PPI group sustainability.

All the participants in the workshops were subsequently invited to take part in individual interviews to talk about their motivations and experience of taking part in the project and their thoughts on how the newly formed PPI group could be sustained in the future. The issue of anonymity was not applicable to the workshops as the participants got to know one another during the course of the workshops which were held on Zoom. Thirteen semi-structured interviews were undertaken with six patients, five family members (4 of these bereaved), and two coordinators from mesothelioma/asbestos patient organisations. The interviews were conducted via Zoom, each lasting approximately 35–45 min, transcribed anonymously, and analysed using Thematic Analysis [30] whereby the themes represented the main patterns of meaning in the data. The analysis was largely inductive in that the coding and the theme development were driven by the data content rather than existing concepts or theories. The NVivo12 software was used to facilitate the organisation of the data analysis [31]. The first author led on the data analysis and followed the six phases of Thematic Analysis. The first author read the transcripts and familiarized herself with the dataset, then generated codes (succinct labels) that were applied to the transcripts. In the third phase initial themes were generated by examining commonalities between the codes and observing the emergence of wider patterns of meaning, and in the fourth phase themes were constructed and reviewed with input from two other members of the research team. The themes were subsequently refined and the detailed analysis of each theme was written up. All data were stored securely on the project folder held on the university server, to which only the research team had accessed.

## Results

### Study 1: the researchers’ perspective on PPI in mesothelioma

Twenty-three participants took part in the pilot and main survey, of which 26% (n = 6) indicated that they did not currently include PPI in their research, while the remaining 74% (n = 17) did, see full features of the participants in Table 2.

**Table 2:** Features of the mesothelioma researchers participating in the survey (n = 23)

**Gender**	
Female	11 (48%)
Male	12 (52%)
*Professional role*	
Academic	12 (52%)
Clinical researcher	5 (22%)
Clinical academic researcher	5 (22%)
Research support role	1 (4%)
*Main type of research*	
Basic	6 (26%)
Translational	4 (18%)
Clinical	8 (34%)
Psychosocial	4 (18%)
Other	1 (4%)
*Range of current mesothelioma research*	
Clinical trial	7 (30%)
Cohort study	5 (22%)
Psychosocial intervention aimed at patients	2 (9%)
Psychosocial intervention aimed at carers/family members	3 (13%)
Other *(genomics; patient stratification; drug discovery and mechanism of* *mesothelioma; drug discovery and development; basic science; translational research mostly using mesothelioma cell lines or tumour specimens retrospectively; biomedical; qualitative interview study; survey of early symptom experience; health services research and patient experience research)*	12 (52%)

Among the six respondents who did not include PPI, four were academics (i.e. teachers or scholars in universities or other institutes of higher education) and two were clinical researchers. Three were conducting basic, two, translational, and one, clinical research. Some indicated that the type of research they currently conducted on mesothelioma comprised genomics, patient stratification, and biomedical research. Seven items derived from research on PPI aimed to capture the most common reasons for not including PPI in mesothelioma research, e.g. finding it difficult to recruit patients or public as PPI representatives or being unsure how to include PPI, see Table 3.

**Table 3:** Reasons for not including PPI in mesothelioma research (n = 6)

Reasons	Frequency
I find it difficult to recruit patients and/or public to include as PPI in my research	3 (50%)
I am unsure/don’t know how to include PPI	3 (50%)
I do not have capacity among my team to include PPI	2 (33.3%)
PPI is of little benefit to my research	2 (33.3%)
The funders I usually apply to do not require PPI	1 (16.6%)
PPI is not applicable to my research	1 (16.6%)
I prefer not to include PPI in my research because of its costs and challenges	0
Other *At the time of funding PPI was not a requirement*	1 (16.6%)

Most of the six MRN members who did not include PPI in their research indicated that PPI might be beneficial at the later stages of the research, such as disseminating the findings (83.3%, n = 5) and facilitating the uptake of research findings (66.6%, n = 4). As for the perceived costs of PPI in mesothelioma research, the six MRN members agreed that PPI would increase both costs (66.6%, n = 4) and time (66.6%, n = 4) for researchers and institutions due to the practical aspects of planning and managing PPI.

We next examined the responses of the researchers (n = 17) who included PPI in their mesothelioma research at the time of the survey. Overall, their professions ranged from academic (n = 8), to clinical researcher (n = 4), clinical academic (i.e. clinical professionals combining a clinical career with a research career, working across healthcare providers and academic institutions) (n = 4) and research support role (n = 1). Their main type of research was basic (n = 3), translational (n = 2), clinical (n = 5), psychosocial (n = 3), patient experience and health services research (n = 1). The range of research they were conducting included: clinical trial (n = 6), cohort study (n = 4), psychosocial intervention aimed at patients (n = 3), and other (n = 8), such as: drug discovery and mechanism of mesothelioma; drug discovery and development; translational research mostly using mesothelioma cell lines or tumour specimens retrospectively; qualitative interview study; survey of early symptom experience; health services research and patient experience research. The respondents usually involved patients, carers and members of mesothelioma / asbestos-disease charities in their PPI activities, see Table 4.

**Table 4:** Groups typically included as PPI representatives by mesothelioma researchers (n = 17)

People included as PPI representatives	Usually	Sometimes	Never	N/A
Members of mesothelioma or asbestos-related disease charities / support groups	10 (59%)	4 (24%)	3 (17.6%)	0
Patients undergoing curative or palliative treatment	10 (59%)	4 (24%)	2 (11.7)	1 (6%)
Informal carers (family or friends)	10 (59%)	4 (24%)	2 (11.7)	1 (6%)
Newly diagnosed patients	9 (53%)	2 (11.7)	5 (29%)	1 (6%)
Healthcare staff specialising in mesothelioma	9 (53%)	5 (29%)	3 (17.6%)	0
Healthcare staff specialising in lung cancer	8 (47%)	5 (29%)	4 (24%)	0
Bereaved family or friends	7 (41%)	3 (17.6%)	6 (35%)	1 (6%)
Healthcare staff specialising in cancer	7 (41%)	3 (17.6%)	7 (41%)	0

The 17 respondents who included PPI in their research indicated that they did so usually at the stages of: disseminating the findings (82%, n = 14), facilitating the uptake of research findings (65%, n = 11), applying for funding (65%, n = 11), and at the stage of study design and procedures (65%, n = 11). The MRN respondents who typically included PPI had found this to be beneficial for their mesothelioma research and believed their research would benefit from including more PPI than it currently did: *yes* (82%, 14), *no* (6%, n = 1), *not sure* (11.7%, n = 2). They agreed that PPI was most beneficial at the stages of applying for funding, dissemination, and facilitation of uptake of research findings, see Table 5. The main benefits of PPI were clarifying the research question and outcome measures, making research more credible and relevant to the patients’ needs, and increasing its impact, see Box 1 below for a selection of their comments. Finally, the MRN members who typically included PPI reflected, in open text, on the main drawbacks and challenges when including PPI in their research. Some mentioned the practical barriers of including PPI, such as lack of funding for PPI activities and tight deadlines for research proposal submission, while others mentioned that the nature of the disease with high symptom burden and short prognosis limited the scope for PPI inclusion from the start to the end of research projects. In Box 2 we include a selection of participants’ comments.

**Table 5:** Stage of research where PPI is perceived as most beneficial (n = 17)

Stages of the research process	Beneficial	Neither beneficial nor unhelpful	Not beneficial	Don’t know, N/A
Dissemination	13 (76%)	4 (24%)	0	0
Facilitating uptake of research findings	11 (65%)	5 (29%)	1 (6%)	0
Applying for funding	11 (65%)	3 (17.6%)	1 (6%)	0
Recruitment of study participants	9 (53%)	3 (17.6%)	4 (24%)	1 (6%)
Priority setting	7 (41%)	2 (11.7)	5 (29%)	3 (17.6%)
Study design and procedures	6 (35%)	4 (24%)	5 (29%)	2 (11.7)
Data analysis	5 (29%)	3 (17.6%)	7 (41%)	2 (11.7)
Data collection	4 (24%)	4 (24%)	7 (41%)	2 (11.7)

Box 1 MRN members’ experiences of benefits of including PPI in mesothelioma research (n = 17)

- *My research goal is better defined with PPI/E. Knowing that my research might bring about beneficial outcome to patients and the public, I would feel more motivated*. (academic, translational research)
- *Gaining a patient and carers perspective always adds a new perspective on findings and validates if they feel they reflect their experiences. They also loved to be asked and like to feel they are adding something back which may benefit others with the disease*. (researcher in a support role, psychosocial research)
- *Keeps the research relevant to key issues facing patients and relatives such as access to treatment*. (academic, basic research)
- *Keeps you grounded in reality and why we do research*. (academic, basic research)
- *Enables clarity in explanation or project to lay public and participants. Discussions with patients and families at the June Hancock mesothelioma research events is important in ensuring my scientific colleagues appreciate the value of their work. We find this motivating*. (academic, respiratory medicine, basic research)
- *Massively. For the multicentre trial, the research question was developed by the James Lind Alliance Priority Setting Partnership brought about the research question. We have a patient with mesothelioma on the TMG [trial management group] so has significant input into all aspects of the trial. I am designing a new mesothelioma trial and our trial representative has contacted patients in her support group to review the proposed trial design. We have multiple patient representatives on the Trial Review Group so ask the questions that doctors and scientists would not think to ask at the very early stages*. (academic, senior research fellow, psychosocial research)
- *The determination of relevant outcome measure. The production of patient-centred study documentation, particularly patient information sheets*. (academic, researcher in mesothelioma, psychosocial research)
- *Improve relevance, uptake and impact*. (academic, mesothelioma and nursing research, patient experience and health services research)
- *Strengthening grant applications, informing researchers of issues important to patients*. (clinical researcher, Consultant Thoracic Surgeon)
- *PPI/E is always valuable and can make the research more credible*. (clinical researcher, Mesothelioma UK CNS)

## Box 2 MRN members’ experiences of the challenges of including PPI in mesothelioma research (n = 17)

- *Confidentiality of results. Telling patients a lack of funding is preventing more rapid progress.* (clinical academic, senior trial manager, clinical research)
- *Difficult to find mesothelioma patients who are keen to participate in PPI and who are well enough to participate over the long period of time it takes to develop and run a clinical trial.* (clinical academic, clinical oncology research)
- *Given the nature of the disease finding an appropriate time to approach people about PPI/E can be challenging and those able to see a research project all the way through are limited*. (researcher in a support role, psychosocial research)
- *One challenge is engaging the community affected by mesothelioma in research*. (academic, basic research)
- *Rapidly identifying willing participants*. (academic, respiratory medicine, basic research)
- *Personally I am always concerned about the health of the patient rep and whether it is appropriate to contact them*. (academic, psychosocial research)
- *The main drawback to PPI/E in mesothelioma research is continuity; poor prognosis and short survival are limiting factors especially in grant applications which can be lengthy processes*. (academic, psychosocial research)
- *Funding timescales sometimes make it hard* (academic, patient experience and health services research)
- *With mesothelioma patients, finding patients with a sufficiently long disease free interval to contribute for a long enough time for it to be beneficial*. (clinical researcher, Consultant Thoracic Surgeon)
- *Ensuring full understanding of the research. Making sure the agenda and proposal is adhered to*. (clinical researcher, Mesothelioma UK CNS)

### Study 2: the patient and public perspective on PPI in mesothelioma

Thirteen semi-structured interviews were undertaken with six patients, five family members (4 of these, bereaved), and two coordinators from mesothelioma patient organisations, and were analysed using Thematic Analysis [30].

Three main themes were developed that summarized the participants’ reflections on their participation in the project, their journey in learning about PPI, and their views on how PPI in mesothelioma could be sustained: “Motivations to become a PPI representative in the future”, “Understanding the nature of PPI during the project”, and “Perceived challenges to PPI in mesothelioma”. In Additional file 1 we provide additional quotes per theme.

### Motivations to become a PPI representative in the future

Wanting to help other mesothelioma patients was one of the main motivations to take part in the project. Some participants also expressed an interest to help mesothelioma researchers:

Now, 5 years down the line, I feel I’ve got a bit more time and I should be doing something towards helping other patients that have been diagnosed. (Interview 2, female patient)

The participants, particularly the patients and their family, were also motivated to get involved in order to find out about ongoing mesothelioma research, trials and treatments:

I will become involved in anything because I feel that the more involved we’ve got, the more information we’ve got. […] Having that information from different people, whether it’s researchers or medical professionals, I think is important. (Interview 3, family member)

The coordinators from the mesothelioma patient organisations were interested to know more about PPI, research and mesothelioma-related activities so that they could inform the patients that they were supporting:

I was just intrigued in the first instance to find out what it was all about really and to see if we could pass that on and try and get more patients involved. (Interview 10, coordinator)

One prominent motivation for participation was the search for meaning and hope for patients and their families. The participants recognized that mesothelioma is difficult to treat but hoped that by getting involved in research they would get access to information about clinical trials which would give them hope about successful treatments:

You do get to hear a little bit more and it gives you a lot of hope. When I was first diagnosed, everything seemed all doom and gloom, we didn’t really know what was going on in the background and I think in the past 5 years a lot more has taken off. If you get to either talk to these people on different studies or see a paper, at least it gives you a bit of positivity. (Interview 2, female patient)

### Understanding the nature of PPI during the project

Many participants were new to PPI and initially they did not know what to expect of the project or of their role as PPI members, either because they were unfamiliar with the concept of PPI or because the project aim was not well explained or easy to grasp. For many participants, the purpose of PPI only became clear with time:

It took me a while to work out what it really was you wanted. (Interview 5, female patient).

It took me a little while to get my head round it and to understand what was expected of people and what it was, what the outcome was going to be. (Interview 11, coordinator)

I think it got better as it went along because to start with I was a little bit confused, but once I realised it was more about the studies and interacting with the professional side of things, I was quite happy. (Interview 2, female patient)

During the course of the project, the concept of PPI became more concrete and the presentations from mesothelioma researchers helped crystallise the notion of PPI, and in some cases helped increase participants’ self-confidence as patient representatives:

I feel completely different about PPI. […] It never meant anything in there to me, whereas now it does. It’s this that’s done it for me. (Interview 4, bereaved family member)

The only thing is [laughs], well I haven’t got a very high level of education and like you were saying about some of the studies, the language and things [researchers] use, I’m not really au fait with it, sometimes I get a little lost. […] As you interact a little bit more with researchers and other medical people, it makes you feel a little bit easier. I’m still nervous about what I say but… Because obviously I’ve started doing one or two studies, I find it slightly easier to talk. (Interview 2, female patient)

However, even among some participants already familiar with PPI there was a sense that one’s understanding of PPI and its role in research can change over time as they understand research better and the speed at which new scientific discoveries can be made.

### Perceived challenges to PPI in mesothelioma

Mirroring the views expressed by the mesothelioma researchers in the online survey, the workshop participants identified a number of practical barriers to becoming involved as PPI representatives in the future due to the nature of mesothelioma, its high symptom burden, and short prognosis after diagnosis. One of our participants expressed how important it is for patients to know their capabilities and vulnerabilities at different times during the illness but especially in the early stages of the illness:

When you’re initially diagnosed, talking about this with other people and thinking, ‘It’s only 18 months,’ it’s something that obviously when you’re told something like that you don’t want to be involved with lots of research and stuff because you feel your time is so precious. (Interview 3, family member)

Furthermore, some participants noted that it is usually the patients who have responded well to treatment that are most likely to volunteer as PPI representatives. Indeed, in our study, most of the people living with mesothelioma had had relatively good responses to treatment and a longer survival after diagnosis than it is usually expected:

Some people can be lucky in the respect that treatments work for them and they are living good quality lives after having these treatments and I think those are the people that really want to help in the progression [of] the research. (Interview 11, coordinator)

However, in the participants’ view, one of the main challenges to becoming a PPI representative was that PPI can be too abstract and difficult to grasp for lay people, particularly when it requires involvement in research which is not necessarily concerned with novel drugs or treatments:

We often say, to us, research is drugs and sorting out which drug to use. But there's a whole new thing about research behind it that isn't about the drugs, it’s about how to do things, how to proceed, how to get information. It took us a while to get used to that, so yes, new patients and carers wouldn't understand that. They want research to be about drugs. (Interview 1, female patient)

The use of scientific language was an additional barrier to becoming involved in PPI activities, with some participants finding scientific jargon rather intimidating. There was a commonly voiced concern among them that they could not match researchers’ scientific knowledge and thus be in any way useful to the research process:

The doctors and the experts start to talk in scientific speak, or doctor speak, and the language is not always everyday language. A couple of things we did pick…that some of the experts were sometimes picked up—“What does that mean?” (Interview 8, male patient)

For the participant living with peritoneal mesothelioma (the only one in our study) an additional challenge was the uniqueness of the disease and its associated lack of treatment, which left the participant unsure as to how she might contribute to research which typically focuses on pleural mesothelioma:

I'm not sure how I could help really. Not having had treatment, I just want to bang the drum really for peritoneal so it gets out there a little bit more. (Interview 5, female patient)

Some bereaved family members viewed their potential future involvement as PPI representatives as less valuable because, for them, the perspectives of people living with mesothelioma should have precedence as they are the direct beneficiaries of research:

It’s a slightly tricky one, isn't it? If you’ve already been there and they're all discussing their particular illnesses and treatments, which is something that’s more difficult for us to get involved with, because it’s not going to benefit us, we can't really get too involved in it. (Interview 12, bereaved family member)

## Discussion

The aims of the project were to explore the barriers and facilitators to PPI in mesothelioma, and to develop best practice for PPI in mesothelioma in collaboration with patients, carers, and patient organisations. We examined the perspectives of mesothelioma researchers via an online survey and the perspectives of patients, carers and the public through online workshops and individual interviews. The survey results indicated that the researchers who usually included PPI had found that this brought benefits to their mesothelioma research, such as clarifying the research question and outcome measures, strengthening grant applications, informing researchers of issues important to patients, making research more credible and relevant to the patients’ needs and increasing its impact. The researchers identified as main barriers to PPI the patients’ poor prognosis and the limited scope for them to be involved for the whole duration of a project. The short prognosis of people living with mesothelioma was not seen as compatible with short funding and research timescales which can prevent timely recruitment of PPI representatives. Furthermore, the lengths of projects were also perceived as a barrier (although in our project most participants stayed with us till the end). The nature of research funding was seen by the surveyed researchers as a barrier to PPI activities, something noted in other studies on health researchers’ experience of PPI [32].

The first theme, *Motivations to become a PPI representative in the future,* developed from the interviews with patients, carers, and coordinators of patient organisations, showed that the participants found the workshops motivating as the project gave them the opportunity to get involved and learn more about mesothelioma research, but also to reduce the sense of loneliness caused by being diagnosed with a rare and incurable disease. Similarly to other studies which have explored the experience of cancer service users involved in PPI activities, our participants were mainly motivated by a desire to get answers to questions around trials and treatment [18], but also to gain a sense of existential meaning and hope for the future [11]. One other notable motivation was altruism, evident in their wish to make things better for others by supporting research and enhancing outcomes for other patients; altruism is an important aspect of the PPI experience as it can impact on contributors’ sense of self and identity [33]. Altruism was also noted by one mesothelioma researcher in the survey who commented that patients and carers like to feel that they are adding something which may benefit others with mesothelioma. Furthermore, developing new research contacts and networks within the workshops gave our participants hope and this hope came about through talking about different studies and sharing information which provided ‘a bit of positivity’ and perhaps counteracted the more negative stories surrounding a diagnosis of mesothelioma [8]. Thus, our participants’ main motivations for participating in the project and in PPI in the future were both social and personal in nature, as noted by other research on PPI [34].

The second theme, *Understanding the nature of PPI during the project*, encapsulated the participants’ gradual understanding of the concept of PPI, their growing sense of confidence and appreciation of the contribution they can make to mesothelioma research. The coordinators of mesothelioma patient organisations understood better during the course of the project that they can play a role in facilitating patients’ and family members’ understanding of PPI activities and participation in research opportunities. The fact that the lay understanding of PPI is a process that requires time has been documented in other PPI studies [18], and reflects the complexity of PPI and the need for sustained training and direct involvement. During the project, the lay participants developed a better understanding of the PPI process through activities such as online modules and researcher presentations at the workshops which facilitated their understanding of PPI. Other lay participants struggled to understand what was wanted and expected of them in the workshops. They also reported feeling unsure of how to work with academics and the specialised language that is used in research and medicine [35]. A turning point happened in workshop 4 when mesothelioma researchers came in to talk to the group about their research. This workshop enabled a dialogue to be opened and relationships to be formed with the researchers and the group. In their work, Smith et al. [36] also reframed involvement as relationships and personalisation within social contexts, and found that service users had a need for more information on roles and relationships before their first meeting. In this project we also needed to balance the needs of the group when developing the agenda for our workshops with the need to provide more information about roles and relationships prior to our first meeting, as a lack of understanding about PPI was reportedly confusing our participants.

Regarding the third theme, *Perceived challenges to PPI in mesothelioma*, we found that PPI can be challenging for researchers as well as patients and carers. Many of our patient participants talked about how hard it is to be involved in research early on in the illness, especially following the shock of the diagnosis and knowledge of the short prognosis and remarked that it was those with a good quality of life, who responded well to treatment, who usually have the time and motivation for involvement in research. In this sense, the patients’ views and experiences around PPI echoed the mesothelioma researchers’ reflections on the challenges associated with PPI. It was striking that some bereaved family members thought that their input into PPI activities is less valuable than that of people diagnosed and living with mesothelioma because they are not the direct beneficiaries of research. This may indicate lack of awareness that mesothelioma research addresses family members’ (or carers’) needs, too [37], on reflection, something that the research team could have emphasised.

In discussing effective user involvement, Tritter and Callum [38] talk about the agency of users who want different ways to involvement. For example, they identify the need for processes of involvement to be dynamic and negotiated by the users themselves and go on to identify how PPI is better conceptualised as the effectiveness of relationships. Therefore, similarly to the finding of previous research on PPI in mesothelioma [35] we found it was possible to involve patients and family members safely in mesothelioma research despite worries about the low survival rate for the illness. Perhaps, following Tritter and Callum, what is needed is a variety of methods of involvement that bring people together at different points of their lives and different points of the mesothelioma illness and care pathway to ensure relevant involvement, guided by the UK Standards for Public Involvement [39]. For example, one-off consultations may suit people living with mesothelioma who are in relatively poor health, while methods requiring multiple meetings, such as co-production, may suit better family members or people living with mesothelioma who are in relatively good health.

### Strengths and limitations

One strength of this study was the ability to recruit patients, family caregivers, bereaved family members and coordinators of mesothelioma / asbestos patient organisations, which enabled us to represent a range of views and experiences. Despite the concerns about the short prognosis of those with mesothelioma and despite unfamiliarity with PPI, we found that recruiting participants through the asbestos support groups and mesothelioma charities and holding our workshops on the Zoom platform enabled us to recruit people affected by mesothelioma from different regions and locations in the country and at different stages of the illness. Furthermore, we recruited patients with different levels of familiarity with—and experience of—PPI which allowed us to discuss the benefits and challenges of PPI from a range of perspectives. This, and the findings from the online survey with mesothelioma researchers, afforded us a comprehensive view of the current challenges and benefits of PPI in mesothelioma research.

Another strength of this study was that the same facilitator ran all the workshops thus providing continuity and a supportive, safe environment for participants. Part of creating a safe space was breaking down barriers during the workshop to get to a position of equality. Developing PPI has also been referred to as creating ‘knowledge spaces’ which facilitate social networks and enable participants to engage on equal terms with professionals [40]. Such spaces are developed to overcome power differences between researchers and PPI participants [41] and enable the PPI participants to bring their real-world perspectives to the group. As noted before, the boundaries between PPI and co-production can sometimes be contentious and unclear, with patient and public partnership in research taking various forms such as collaboration, consultation, participatory action research, or co-design [27, 42, 43]. This was reflected in the present study, where the lines between co-production and PPI were often blurred and the two terms sometimes used interchangeably by the research team. While in our study the patients and the public were mainly ‘data providers’, they were at times also ‘active partners’ [44], roles which can be combined in health research especially in harder-to-reach populations [45]. For example, two research participants, who were bereaved family members, offered input into the design of the workshops in the early stages, while other participants provided feedback on the interpretation of the data through the validation technique of member checking [46]. The two bereaved family members who provided PPI helped with adapting the project from the face-to-face to the online format and advised on how this might work for patients and carers during the COVID-19 pandemic.

Furthermore, conducting the project online enabled us to recruit participants from various locations across the UK and to minimize the burden of research for them, e.g. by removing the necessity to travel. There was high participant retention in our study, and a number of participants attended dissemination events such as Action Mesothelioma Day in July 2021, where some talked about their experience of being involved in this project and learning about PPI. Another strength of our study was the combination of PPI and qualitative research, somewhat similarly to past research [45], which increased the participants’ engagement with the study, improved their understanding of PPI, and helped the research team maximise the contribution of this ‘harder-to-reach’ group. Crucially, this study has had considerable impact, as it has led to the creation of a sustainable PPI group, *Me-So-Involved*, who have since been involved in other research activities (e.g. providing feedback on grant submissions).

There were some limitations to our research, too. First, we were able to recruit and retain mostly people living with mesothelioma who respond well to treatment, and these may not necessarily be representative of mesothelioma patients in general. Second, the requirement to participate in the project online may have reduced our ability to recruit patients less familiar with online technology or may have limited some prospective participants’ motivation to get involved. Also, our sample was not culturally diverse as all of our participants happened to be of White British ethnicity. Last but not least, we had a relatively low response rate to the online survey with mesothelioma researchers which precluded in-depth analysis. It could be that conducting the online survey at the start of the COVID-19 pandemic in 2020 may have limited the participation of mesothelioma researchers as some of these may have been involved in respiratory clinical care at the time.

### Impact

An important outcome of this research project has been the formation of the Me-So-Involved PPI group. In its 1st year of operation, this group, hosted by the June Hancock Mesothelioma Research Fund [47], has been actively involved in a Scottish Medicines Consortium consultation for Nivolumab/Ipilimumab combination immunotherapy as first-line treatment for mesothelioma; a grant application to the JHMRF; and a presentation on patients’ perceptions of care to the first patient and carer session organised by the European Society of Thoracic Surgeons at their annual conference.

### Practical implications

We provide some pointers to good practice to researchers who wish to involve patients and the public in mesothelioma research. This study has resulted in the creation of a new supportive model of PPI for mesothelioma research which will be transferrable to other conditions, to researchers working with rare diseases or with under-served patient groups, and it will be suitable for virtual environments. The study was undertaken during the COVID-19 pandemic, so it was solely virtual; however, as COVID-19 restrictions have eased and mesothelioma support groups meet in person again, the face-to-face monthly meetings provide opportunities for researchers to present their projects and to engage people affected by mesothelioma in PPI activities. As to how PPI in mesothelioma can be made sustainable, this depends on a variety of factors, not least the availability of a platform or venue where researchers and service users can learn about each other’s work and experiences, for example at the ‘Meet the Researcher’ events [21]. Our findings suggest that patients are willing to be involved in a variety of PPI activities but that certain barriers need to be overcome for their involvement to made easier. In Table 6 we detail some recommendations as to how these barriers could be addressed:

**Table 6:** Recommendations to creating effective PPI in mesothelioma research

Challenges	What mesothelioma researchers can do
1. Short timeframes to develop research ideas and apply for grant funding	Engage with local asbestos or mesothelioma support groups, e.g. Hampshire Asbestos Support & Awareness Group (HASAG), to recruit service users interested in research and build long-term collaborations
2. “Patient and public involvement” is hard to grasp as a concept	Draw on the NIHR guidelines on PPI as well as other resources, e.g. Applied Research Collaboration networks, to explain, in lay terms, what PPI means to a lay public. When seeking patients and service users who are new to PPI, give concrete examples of PPI in past research on mesothelioma When involving patients and service users as PPI representatives over the course of a project, designate a research team member as support for PPI representatives in ongoing projects, include them in regular meetings and check if there are aspects of the project that are more difficult to understand. Provide training where necessary and where acceptable to people new to PPI
3. Understanding the concept of “patient and public involvement” is a process that happens over time	Engage with service users over a number of occasions to facilitate their understanding of PPI, e.g. engage with patients and carers at mesothelioma events such as Action Mesothelioma Day Point new PPI representative in the direction of public-facing resources on PPI, e.g. NIHR “Become a Research Champion”, the “Be Part of Research” website, or the “European Patient Ambassador Programme”
4. Abstract nature of PPI and scientific jargon can be intimidating to service users and the lay public	Explain research in lay language, using guidance such as that from NIHR on writing Plain English Summaries. Provide concrete examples of PPI activities to lay public and patients
5. High burden of disease in mesothelioma	Where involving patients is not possible, involve instead family caregivers or bereaved family members and explain to them why their perspective is equally valuable

## Conclusions

To conclude, this research provides a detailed picture of the benefits and challenges of PPI in mesothelioma research and the potential ways in which sustainable PPI could be achieved. We recommend that mesothelioma researchers build relationships with mesothelioma patient organisations to facilitate the understanding of PPI and the involvement of people affected by mesothelioma in research. We also recommend that mesothelioma researchers familiarize themselves with different approaches to PPI, ranging from consultation to co-design, to be able to choose the most appropriate and time-effective method of involvement prior to including PPI representatives in mesothelioma research.

## Supplementary Information

**Additional file1**. Additional quotes for each theme

## Data Availability

Research data are not shared.

## Abbreviations

MRN: Mesothelioma Research Network
PPI: Patient and public involvement
