# Supplementary material for: Developing sustainable patient and public involvement in mesothelioma research: multi-method exploration with researchers, patients, carers, and patient organisations
Source: Res Involv Engagem. 2023 Mar 25;9:15. doi: 10.1186/s40900-023-00426-5 (PMC10039679; doi:10.1186/s40900-023-00426-5)
Supplement: Supplementary file 1 — Additional file1. Additional quotes for each theme [file 40900_2023_426_MOESM1_ESM.docx]

**Additional file 1** Additional quotes for each theme

| **Theme** | **Quotes** |
| --- | --- |
| ***Motivations to become a PPI representative in the future*** | *I’m more than happy to try and help anyone. Obviously, it depends on what’s going on in my life, but anyone can ask me at any time about anything as far as helping in research, I would be more than happy*. (Interview 8, male patient) |
|  | *And it’s even more important when you consider the prognosis, so anything that enhances outcomes for the patient during that time is so important because they don’t have much*. (Interview 4, bereaved family member) |
| ***Understanding the nature of PPI during the project*** | *I didn’t really know what to expect, if I was honest. The whole idea of patient participation involvement, it’s a really great idea. As you have said in the study, things happen about the patient without them having any input, so I think it was a good thing. At first, I kind of struggled in thinking well I’m not a patient, but I did realise that we are a voice for patients in general and we have got that way of getting information out to patients.* (Interview 10, coordinator) |
|  | *I think maybe initially we didn’t quite understand what it was going to be. I thought it was going to be developing, I don't know, like a small community with people that are diagnosed. […] That’s why initially we sort of went forward for it, I believe. With the way it’s gone, I'm not saying that it was a waste of time or we regret doing it, because totally it wasn’t, but it wasn’t quite what we expected. So I suppose the short answer is we thought we might be able to give something to other people*. (Interview 3, family member) |
|  | *When the [researchers] came on and they had their input and they were talking, I think that really helped put into perspective what was happening and what was expected*. (Interview 11, coordinator) |
|  | *To make a group of people, specific to mesothelioma, I think it’s a good idea because you do get a more specific idea from the patients that you are talking to, the people who have the actual illness.* (Interview 8, male patient) |
|  | *I suppose I couldn’t quite see my role for a little bit, but then I sort of settled into it.* (Interview 10, coordinator) |
| ***Perceived challenges to PPI in mesothelioma*** | *When I was first diagnosed, I didn’t want to know about anything like that, too busy coping with the diagnosis.* (Interview 2, female patient) |
|  | *You tend to get more bereaved families involved afterwards because they partly have got time, I suppose, that they would have spent with their partner, and partly that they want to fight now that they’ve lost someone*. (Interview 10, coordinator) |
|  | *A good challenge, not a negative challenge, was actually understanding PPI. So that was a good challenge for me, because even though I had heard those words many times over many years, it never made any impact on me whatsoever.* (Interview 4, family member) |
|  | *It’s not a disease that has a star. Dare I say it, [meso] is an unfashionable disease, they think it’s just a few old people who’ve worked in an asbestos factory, they don’t realise it’s actually affecting so many people, not just old-age pensioners who are at the end of their life anyway. That is part of the trouble, it’s not a fashionable disease.* (Participant 13, workshop 5, bereaved family member) |
